# Fifty high-content light sheet fluorescence microscopy datasets of *Tribolium castaneum* embryogenesis

**DOI:** 10.1038/s41597-025-06406-6

**Published:** 2025-12-15

**Authors:** Frederic Strobl, Pinelopi Goumenaki, Kristina Mirkes, Henrik Tonner, Mariia Golden, Julia Ratke, Franziska Krämer, Stefan Münster, Ernst H. K. Stelzer

**Affiliations:** 1 Physical Biology / Physikalische Biologie (IZN, FB 15), Buchmann Institute for Molecular Life Sciences (BMLS), Cluster of Excellence Frankfurt – Macromolecular Complexes (CEF – MC), Goethe-Universität – Frankfurt am Main (Campus Riedberg), Max-von-Laue-Straße 15, D-60438 Frankfurt am Main, Germany; 2 Philipps-Universität Marburg, Fachbereich Biologie, Molekulare Tierphysiologie, Marburg, Germany

**Keywords:** Embryogenesis, Morphogenesis, Transgenic organisms, Model invertebrates

## Abstract

The red flour beetle (*Tribolium castaneum*) is a key model organism in developmental biology, genetics, and agricultural research. To address the limited availability of high-quality microscopy data documenting its embryonic morphogenesis, we assembled the first Systematic Live Imaging Collection of Embryogenesis (SLICE-1), a large open-access resource for *Tribolium* embryogenesis. Using light sheet fluorescence microscopy, we acquired fifty high-content datasets totaling 200 days of recording time, 5.7 million images, and 2.7 Terabytes of data. Imaging employed five novel transgenic lines expressing mEmerald-labeled nanobodies that target distinct intracellular structures under control of the consecutively and ubiquitously active *tubulin alpha 1-like protein* promoter. Each line encompasses multiple sublines representing independent genomic insertions of the same transgene. Embryos were imaged in triplicates under standardized conditions, and all but three specimens developed into healthy, fertile adults, confirming the minimal invasiveness of the experimental procedure. SLICE-1 enables quantitative and comparative analyses of embryonic development, provides an overview of nanobody functionality in insects, and serves as a training resource for image processing, machine learning, and neural networks.

## Background & Summary

Developmental biology seeks to understand how a single cell gives rise to a fully functional multicellular organism, uncovering fundamental principles that orchestrate cell migration, proliferation, and differentiation^1^. Three-dimensional imaging techniques have become a cornerstone methodology for examining embryonic morphogenesis from the subcellular to the *in-toto* level^2^. In particular, light sheet fluorescence microscopy (LSFM) is well-suited for long-term live imaging due to its high signal-to-noise ratio, fast recording speed, deep tissue penetration, and low photobleaching/phototoxicity^3^. Microscopy-based experiments typically follow one of two strategies: (i) an on-demand approach, which addresses specific scientific questions, or (ii) a systematic approach, which generates comprehensive, multi-dimensional datasets intended as reusable resources for large, interdisciplinary research communities^4^. In particular, systematically acquired morphogenetic data serve both as convergence points that enable the chronological alignment of on-demand data across a universal developmental framework, and as divergence points that provide the foundation for uncovering previously unrecognized morphogenetic processes or formulating hypotheses about underlying biological mechanisms. Beyond developmental biology, such data are valuable to a range of adjacent fields. Biophysicists use image data to infer biomechanical forces during morphogenesis^5,6^, while laboratories focused on a single model organism employ such information to embed their findings in a broader evolutionary context^7^. Additionally, the performance of machine learning- and artificial intelligence-based image analyses improves with increasing volume and diversity of training data^8^.

While ample imaging data are available for the zebrafish (*Danio rerio*) and the fruit fly (*Drosophila melanogaster*) in open-access databases such as Zenodo^9^, Image Data Resource (IDR)^10^ and BioImage Archive^11^, comparable resources remain scarce for emerging model organisms like the red flour beetle (*Tribolium castaneum*). Recognized as a valuable complement for studying both conserved and divergent aspects of insect development^12^, *Tribolium* offers several advantages: genetic tractability^13,14^, a key phylogenic position within the Holometabola^15^, and a slower, seemingly more ancestral mode of embryogenesis^16^. However, the limited availability of microscopy data restricts its broader use in quantitative and comparative research. Expanding the number of openly accessible datasets is therefore essential to complement its well-established genetic toolkit^17–20^ with comprehensive morphogenetic context. This will not only strengthen the interface between the molecular, cellular, tissue and organismal levels, but also introduce a second analytical axis for comparison, thereby fostering integrative and evolutionary informed analyses.

In this data descriptor, we present the first Systematic Live Imaging Collection of Embryogenesis (SLICE-1) comprising fifty high-content fluorescence microscopy datasets of *Tribolium castaneum* embryonic development, totaling 200 days of recording time, 5.7 million images, and 2.7 Terabytes of data (Supplementary Table 1). Datasets were acquired using LSFM in conjunction with five novel transgenic lines specifically designed for long-term fluorescence live imaging. Each line encompasses multiple sublines representing distinct genomic insertions of the same transgene. To specifically label intracellular structures, we assayed the functionality of five nanobodies^21^, which are derived from vertebrate antigens, in *Tribolium*. The nanobodies target (i) cytokeratin-8 (i.e. the non-binding nanobody control), (ii) histone H2A/H2B, (iii) proliferating cell nuclear antigen (PCNA), (iv) lamin A/C, and (v) actin. Imaging began at the end of blastoderm formation and continued for either ~24 hours (covering gastrulation and part of germband elongation) or ~120 hours (covering gastrulation, germband elongation, germband retraction, dorsal closure, and part of muscular movement). Each specimen was recorded along four orientations spaced 90° apart, ensuring the availability of ventral, lateral, and dorsal (or ventrolateral and dorsolateral) views, and enabling central downstream processing approaches such as multiview fusion for the generation of superior representations of the data^22^. All but one embryo hatched morphologically intact, all remaining but one larva developed into healthy adults, and all remaining but one adult were fertile. Raw data were pre-processed (alignment of the anterior-posterior axes of the embryos with the *y* axis of the images followed by cropping in all three spatial dimensions to remove non-informative background and reduce data volume) and *z* maximum projections were calculated. Five of the assayed sublines displayed atypical fluorescence signal, likely attributable to position effects arising from variation in transgene insertion sites.

## Methods

### *Tribolium castaneum* strains and rearing

For the creation of novel transgenic lines, the *Tribolium castaneum* Plain-White-As-Snow background strain^23^ was used. *Tribolium* cultures were kept in sizes of about 200–500 individuals on growth medium (full grain wheat flour (SP061036, Demeter) supplemented with 5% (wt/wt) inactive dry yeast (62–106, Flystuff)) in one-liter glass bottles in a 12:00 h light / 12:00 h darkness cycle either at 32 °C or at 25 °C and 70% relative humidity (DR-36VL, Percival Scientific). Single pair crossings were performed in small glass vials filled with 1.5–2.5 g growth medium. All animal-related experiments were approved by the institutional ethics committee of the Goethe University (Tierschutzkommission der Goethe-Universität, file number BB-20250515-St) and conducted in agreement with the German Animal Welfare Act (Tierschutzgesetz/Tierschutz-Versuchstierordnung, based on ETS No.123^24^ and EU Directive 2010/63/EU^25^) as well as the ARRIVE 2.0 guidelines^26^.

### Plasmids, germline transformation and transgenic lines / sublines

Based on commercially available nanobody-encoding plasmids (cat. nos. tcg/ccr/lcg/acg, ChromoTek/Proteintech), *Tribolium*-optimized coding sequences for nanobodies against (i) cytokeratin-8^21^, (ii) histone H2A/H2B^27^, (iii) PCNA^21^, (iv) lamin A/C^21^, and (v) actin^28^ were *de-novo* synthetized (GeneArt, Thermo Fisher Scientific), amplified by PCR and inserted into the #O slot of pAGOC{#P’#O(LA)-mEmerald}^23^, replacing the default Lifeact (LA) coding sequence. Next, for all five intermediates, the endogenous *tubulin alpha 1-like protein* promoter^29^, which is constitutively and ubiquitously active, was inserted scarlessly into the #P slot. The five resulting transformation-ready plasmids (Table 1, ‘Plasmid’ column) were separately injected into F1 pre-blastoderm embryos to achieve germline transformation using the previously described pATub’piggyBac helper plasmid^23^ as the piggyBac transposase source. All F1 survivors were mated with wild types, and in (i) three, (ii) four, (iii) two, (iv) five, and (v) six crossings (twenty in total), at least one transgenic F2 individual was found among the descendants. All transgenic individuals were mated with wild-type individuals to estimate the number of insertions and, for each F1 crossing that resulted in transgenic progeny, one of the F2 crossing setups that resulted in ~50% transgenic F3 progeny was chosen to found a subline (Table 1, ‘Transgenic line’ and ‘Subline’ columns, and Table 2). Among the F3 progeny, one female per subline was used to start the four-generation AGOC mating procedure for a systematic creation of homozygous cultures as described previously^23^ (Supplementary Table 2). Homozygous F7 individuals were obtained for (i) three, (ii) four, (iii) two, (iv) five, and (v) one of the sublines (fifteen in total, cf. Supplementary Table 2, ‘F6’ row) and used to establish F7+ continuative homozygous cultures. For one (#2) of the five homozygous lethal sublines (all five were actin nanobody-expressing sublines), a mixed culture was established and regularly curated (i.e. wild-type individuals were removed during every culture clean-up routine). In addition to the newly created sublines, the previously described EFA-nGFP line^30^ as well as the AGOC{ATub’H2B-mEmerald} #3 and AGOC{ATub’#O(LA)-mEmerald} #1 sublines^23^ were used.

**Table 1:** Plasmids as well as transgenic *Tribolium* lines and sublines created for or used during this study. In the homozygous viability (‘HzVb’) column, green shading indicates homozygous viable sublines, while red shading indicates homozygous lethal sublines. In the systematic imaging (‘SysImg’) column, green shading indicates sublines for which systematic live imaging was performed in triplicates, yellow shading indicates sublines for which only one dataset was acquired, and red shading indicates that no live imaging data was collected. In the position effect (‘PE’) column, green shading indicates expression-as-expected sublines, while red shading indicates position-effect sublines.

**Table 2:** Insert number estimation assays for all newly created transgenic sublines.

Transgenic line	Subline	F3 marker distribution		
				total
AGOC{ATub’Cyto8°NB-mEmerald}	#1	43.7% (31)	56.3% (40)	71
	#2	49.5% (54)	50.5% (55)	109
	#3	49.4% (78)	50.6% (80)	158
AGOC{ATub’H2A/H2B°NB-mEmerald}	#1	46.7% (43)	53.3% (49)	92
	#2	42.9% (39)	57.1% (52)	91
	#3	50.0% (56)	50.0% (56)	112
	#4	47.6% (49)	52.4% (54)	103
AGOC{ATub’PCNA°NB-mEmerald}	#1	45.0% (36)	55.0% (44)	80
	#2	57.5% (61)	43.5% (47)	108
AGOC{ATub’Lamin°NB-mEmerald}	#1	56.6% (64)	43.3% (49)	113
	#2	56.1% (64)	43.9% (50)	114
	#3	49.6% (56)	50.4% (57)	113
	#4	55.4% (67)	44.6% (54)	121
	#5	49.5% (51)	50.5% (52)	103
AGOC{ATub’Actin°NB-mEmerald}	#1	49.6% (62)	50.4% (63)	125
	#2	51.7% (62)	48.3% (58)	120
	#3	47.4% (46)	52.6% (51)	97
	#4	45.5% (50)	54.5% (60)	110
	#5	39.5% (64)	60.5% (98)	162
	#6	45.5% (51)	54.5% (61)	112

For a detailed description of the insert number estimation assay, please refer to the respective study^23^. Note that only the results from the crossings used to establish sublines are shown, data from discarded crossings are not included.

### Light sheet fluorescence microscopy

Light sheet fluorescence microscopy was implemented using a sample chamber-based digital scanned laser light sheet fluorescence microscope (DSLM)^31^, which generates a dynamic light sheet by rapidly scanning a collimated Gaussian laser beam with a single two-axes piezo-driven scanning mirror (M-116.DG, Physik Instrumente GmbH & Co KG). A 488 nm / 20 mW diode laser (PhoxX 488-20, Omicron Laserprodukte GmbH) with a 488 nm cleanup filter (xX.F488, Omicron Laserprodukte GmbH) served as the illumination light source. Excitation was performed through a 2.5× NA 0.06 EC Epiplan-Neofluar objective (422320-9900-000, Carl Zeiss AG) while the emission was collected through a 10× NA 0.3 W N-Achroplan objective (420947-9900-000, Carl Zeiss AG). A 525/50 single-band bandpass filter (FF03-525/50-25, Semrock/AHF Analysentechnik AG) and a high-resolution charge-coupled device camera (Clara, Andor) were used for detection. Conventionally^3^, the illumination axis is defined as *x*, the rotation axis as *y*, and the detection axis as *z*, with *y* being collinear with Earth’s gravity. Three micro-translation stages (M-111.2DG, Physik Instrumente GmbH & Co KG) and one precision rotation stage (M-116.DG, Physik Instrumente GmbH & Co KG) were used for sample translation along *x*, *y*, and *z* as well as rotation around *y*, respectively. The effective laser power was regularly measured with an optical power and wavelength meter (OMM-6810B and OMH-6703B, Newport).

### Embryo collection, preparation, mounting, live imaging and retrieval

In principle, embryo collection, preparation and mounting were performed as described previously^32,33^. To establish imaging cultures for homozygous viable sublines (cf. Table 1, ‘HzVb’ column), ~300 individuals were taken from the F7+ continuative (mC/mC) homozygous cultures. To establish transient (i.e. one-generation) imaging cultures of a second actin nanobody-expressing subline, ~150 (mC/-) hemizygous virgin females from the AGOC{ATub’Actin°NB-mEmerald} #2 subline were mated with ~150 wild-type males. Between collection and preparation, embryos were incubated at room temperature (23 °C ± 1 °C) for 15 h to reach the end of blastoderm formation. Pre-selection of transgenic (i.e. fluorescent) embryos was not necessary for specimens derived from imaging cultures of homozygous viable sublines, since respective descendants are always homozygous. For embryos derived from transient imaging cultures, which may be wild-type or heterozygous, pre-selection was not performed to avoid harmful light exposure.

In the DSLM, axial image (*z*) stacks of embryos were recorded in one fluorescence channel along four directions in 90° steps and at up to 301 time points with an interval of 30 min (corresponding to a total recording time of up to 150 h). Embryos were illuminated with a laser power of 135 µW during a 50 ms exposure time window of the camera. All *z* stacks have a lateral voxel pitch of 0.645 µm and an axial voxel pitch of 2.58 µm (i.e. a pitch ratio of 1:4). Once imaging was completed, embryos were retrieved from the microscope chamber as described previously^34^.

### Image processing

Image processing was performed using Fiji^35^ (based on ImageJ^36^, Version 1.53f) and *Mathematica* (Version 13.3.0.0)^37^. Firstly, in Fiji, *z* maximum projections were calculated for all *z* stacks and concatenated to time (*t*) stacks. Secondly, using a custom *Mathematica* program (cf. Code Availability section), *z* and *t* stacks of directions in which the embryo was tilted were rotated around *z* to align the anterior-posterior axes of the embryos with the *y* axis of the images. The applied rotations typically ranged between 1° and 5°, with pixels resampled using a Gaussian kernel. During this step, the images were also cropped to a size of 600 × 1000 × 150 voxels, or in the case of large embryos, 600 × 1100 × 150 voxels. Thirdly, in Fiji, the *t* stacks for all directions of a dataset were combined to horizontal *t* stack montages, which were manually inspected to classify sublines as either expression-as-expected sublines or as position-effect sublines, i.e. sublines that exhibit atypical fluorescence patterns caused by the local regulatory landscape of the transgene insertion site (cf. Table 1, ‘PE’ and ‘Comment’ columns). Fourthly, for datasets from expression-as-expected sublines, *t* stack montages were subjected to histogram matching in Fiji (Bleach Correction → Histogram Matching) to equalize the signal intensity over the entire time course and then adjusted in brightness and contrast. Since histogram matching may obscure position effect signals, *t* stack montages for datasets from position-effect sublines were adjusted only in brightness and contrast. Fifthly, the processed *t* stack montages were split up into single direction *t* stacks as well as re-combined to vertical *t* stack montages. Finally, all images were ZIP-compressed based on the TIFF-intrinsic deflate option.

## Data Record

The SLICE-1 datasets are available for download at Zenodo^38^ as ZIP-compressed TIFF files. Depending on dataset volume, files were compiled into a single or split into two or four ZIP archives (cf. Supplementary Table 1, ‘Access’ column). In cases where datasets are split into multiple archives, the first part always contains all *t* stack files. Each dataset encompasses six acquisition dimensions: the first (*x*) and second (*y*) spatial dimensions are obtained simultaneously during one camera acquisition period. The third spatial dimension (*z*) is represented by the optical sections that are recorded while the embryos are moved stepwise through the light sheet. The resulting *z* stacks are stored as single files using the TIFF-intrinsic multi-page function (indicated as PL(ZS) within the file name). Together, the three spatial dimensions define the volume of view. The further dimensions are the fluorescence channel (one), the direction (four) and the time point (up to 301), which are stored as separate files (B2 folder, indicated as CH, DR or TP in the file name, respectively).

**Fig. 1:**
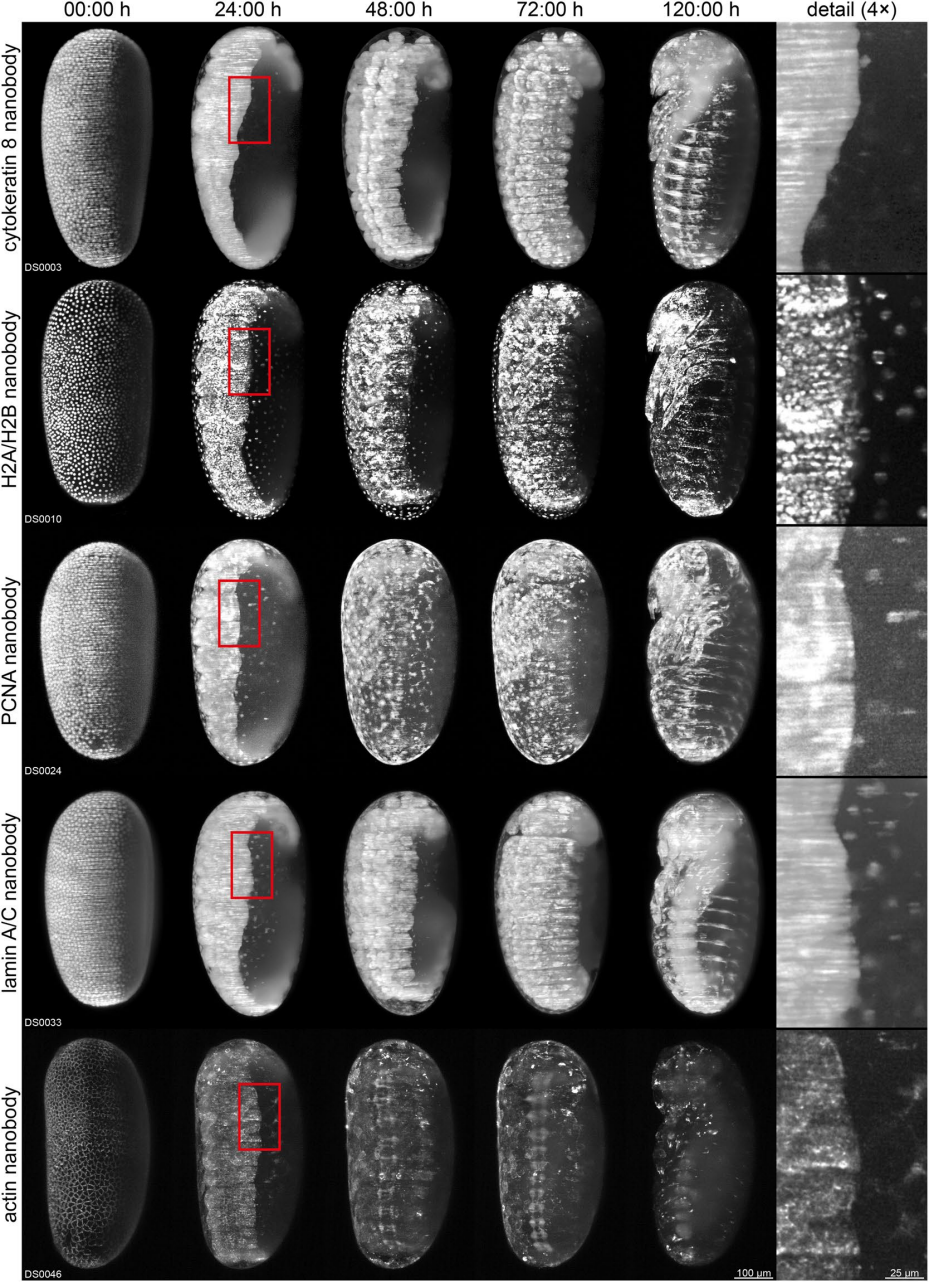
Comparative overview of all five transgenic lines based on image data from expression-as-expected sublines. Shown are *z* maximum projections derived from *z* stacks of embryos from sublines in which the *tubulin alpha 1-like protein* promoter drives the expected ubiquitous and constitutive expression pattern. All embryos are shown in ventrolateral view.

For convenience, *z* maximum projections are provided for each dataset. These are simplifications of the *z* stacks in which one spatial dimension (*z*) is collapsed. Projections are provided as *t* stacks in two versions per dataset, (i) as raw *z* maximum projections (B3 folder, indicated as PL(ZM) within the file name) and (ii) as processed *z* maximum projections, either with histogram matching followed by brightness/contrast adjustments (for datasets from expression-as-expected sublines, indicated as PL(ZH) within the file name) or alternatively *z* maximum projections with only brightness/contrast adjustments (for datasets from position-effect sublines, indicated as PL(ZA) within the file name). Respective *t* stacks are stored as single TIFF multi-page files (B3 and B4 folders, indicated as TP(TS) within the file name), the processed versions are further provided as horizontal and vertical direction montages (B5 folder, indicated as DR(AX) or DR(AY) within the file name, respectively). A comparative overview of all transgenic lines, based on image data from expression-as-expected sublines, is shown in Fig. 1. Each dataset includes comprehensive metadata in form of a human- and machine-readable XLSX file (typically located in the B1 folder, but extracted from the archives for convenient separate download). In addition, each dataset is accompanied by two movie files: a TIFF multi-page file at native resolution (denoted as FQ for ‘full quality’) viewable in e.g. Fiji, and a PNG-compressed AVI downscaled by a factor of two (denoted as HD for ‘high definition-like’) viewable in e.g. the VLC Media Player. These movies are based on the horizontal direction montages and include orientation, time and scale labels.

## Data Overview

For each dataset, depending on the total recording duration, the onset time points of two to five key embryogenetic events were annotated (Table 3): early gastrulation (defined as the initial period of gastrulation lasting until the onset of germband elongation), germband elongation, germband retraction, dorsal closure, and muscular movement (defined as the period from the end of dorsal closure until hatching). These annotations are also listed in the metadata files.

**Table 3:** Embryogenetic events starting time point annotation.

Subline	Dataset	Starting time point				
		EG	GE	GR	DC	MM
**Datasets from sublines expressing mEmerald-labeled nanobodies against cytokeratin-8**						
AGOC{ATub’Cyto8°NB-mEmerald} #1	0001	0010	0026	—	—	—
	0002	0012	0029	—	—	—
	0003	0011	0027	0079	0203	0232
AGOC{ATub’Cyto8°NB-mEmerald} #2	0004	0011	0024	—	—	—
	0005	0011	0028	—	—	—
	0006	0010	0025	0078	0202	0228
AGOC{ATub’Cyto8°NB-mEmerald} #3	0007	0007	0028	—	—	—
	0008	0010	0026	—	—	—
	0009	0009	0025	0073	0191	0220
**Datasets from sublines expressing mEmerald-labeled nanobodies against histone H2A/H2B**						
AGOC{ATub’H2A/H2B°NB-mEmerald} #1	0010	0001	0014	0066	0203	0233
	0011	0001	0015	0073	0218	—
	0012	0004	0019	0062	0185	0211
AGOC{ATub’H2A/H2B°NB-mEmerald} #2	0013	0002	0019	0088	0256	0282
	0014	0001	0016	0069	0216	—
	0015	0001	0011	0065	0195	0231
AGOC{ATub’H2A/H2B°NB-mEmerald} #3	0016	0004	0020	0086	0255	—
	0017	0004	0023	0086	0239	0276
	0018	0002	0020	0084	0237	0277
**Datasets from sublines expressing mEmerald-labeled nanobodies against PCNA**						
AGOC{ATub’PCNA°NB-mEmerald} #1	0019	0007	0022	—	—	—
	0020	0009	0023	—	—	—
	0021	0002	0018	0077	0188	0213
AGOC{ATub’PCNA°NB-mEmerald} #2	0022	0010	0024	0090	—	—
	0023	0009	0026	—	—	—
	0024	0007	0020	0058	0184	0211
**Datasets from sublines expressing mEmerald-labeled nanobodies against lamin A/C**						
AGOC{ATub’Lamin°NB-mEmerald} #1	0025	0013	0026	0070	0174	0200
	0026	0014	0028	0073	0175	0199
	0027	0013	0028	0078	0180	0207
AGOC{ATub’Lamin°NB-mEmerald} #2	0028	0008	0026	—	—	—
	0029	0009	0021	0078	0235	—
	0030	0016	0031	0096	0236	—
AGOC{ATub’Lamin°NB-mEmerald} #3	0031	0011	0028	—	—	—
	0032	0009	0031	unknown^1^	0244	—
	0033	0003	0018	0065	0169	0201
AGOC{ATub’Lamin°NB-mEmerald} #4	0034	0014	0028	—	—	—
	0035	0014	0033	—	—	—
	0036	0013	0034	0107	0226	0257
AGOC{ATub’Lamin°NB-mEmerald} #5	0037	0010	0024	—	—	—
	0038	0011	0027	—	—	—
	0039	0008	0025	0095	0217	—
**Datasets from sublines expressing mEmerald-labeled nanobodies against actin**						
AGOC{ATub’Actin°NB-mEmerald} #2	0040	0010	0028	0086	0232	—
	0041	0003	0016	0069	0212	0249
	0042	0004	0023	0106	0251	—
AGOC{ATub’Actin°NB-mEmerald} #5	0043	0014	0031	0082	0201	0234
	0044	0010	0025	0080	0207	0241
	0045	0008	0019	0061	0155	0183
**Additional datasets from various lines/sublines (controls and peculiarities of development)**						
EFA-nGFP	0046	0004	0018	0083	0230	0262
AGOC{ATub’H2B-mEmerald} #3	0047	0011	0025	0080	0180	0212
AGOC{ATub’#O(LA)-mEmerald} #1	0048	0001	0014	0071	0227	0259
AGOC{ATub’H2A/H2B°NB-mEmerald} #1	0049	unknown^2^	0038	0120	—	—
AGOC{ATub’H2A/H2B°NB-mEmerald} #3	0050	0004	0017	0067	0173	—

Starting time points were identified by the following morphogenetic criteria: early gastrulation (EG), rearrangement of the uniform blastoderm; germband elongation (GE), closure of the serosa window on the ventral side; germband retraction (GR), first sign of movement of the posterior tip of the germband towards the posterior pole; dorsal closure (DC), rupture of the serosa in the anterior-ventral region; muscular movement (MM), completion of germband flank closure.

^1^Due to a slight germband torsion in this embryo (cf. Technical Validation section), the precise starting time point of germband retraction could not be determined. ^2^Due to an atypical proliferation pattern during the 12^th^ synchronous division wave of blastoderm formation in this embryo (cf. Technical Validation section), the precise starting time point of early gastrulation could not be determined.

## Technical Validation

**Fig. 2:**
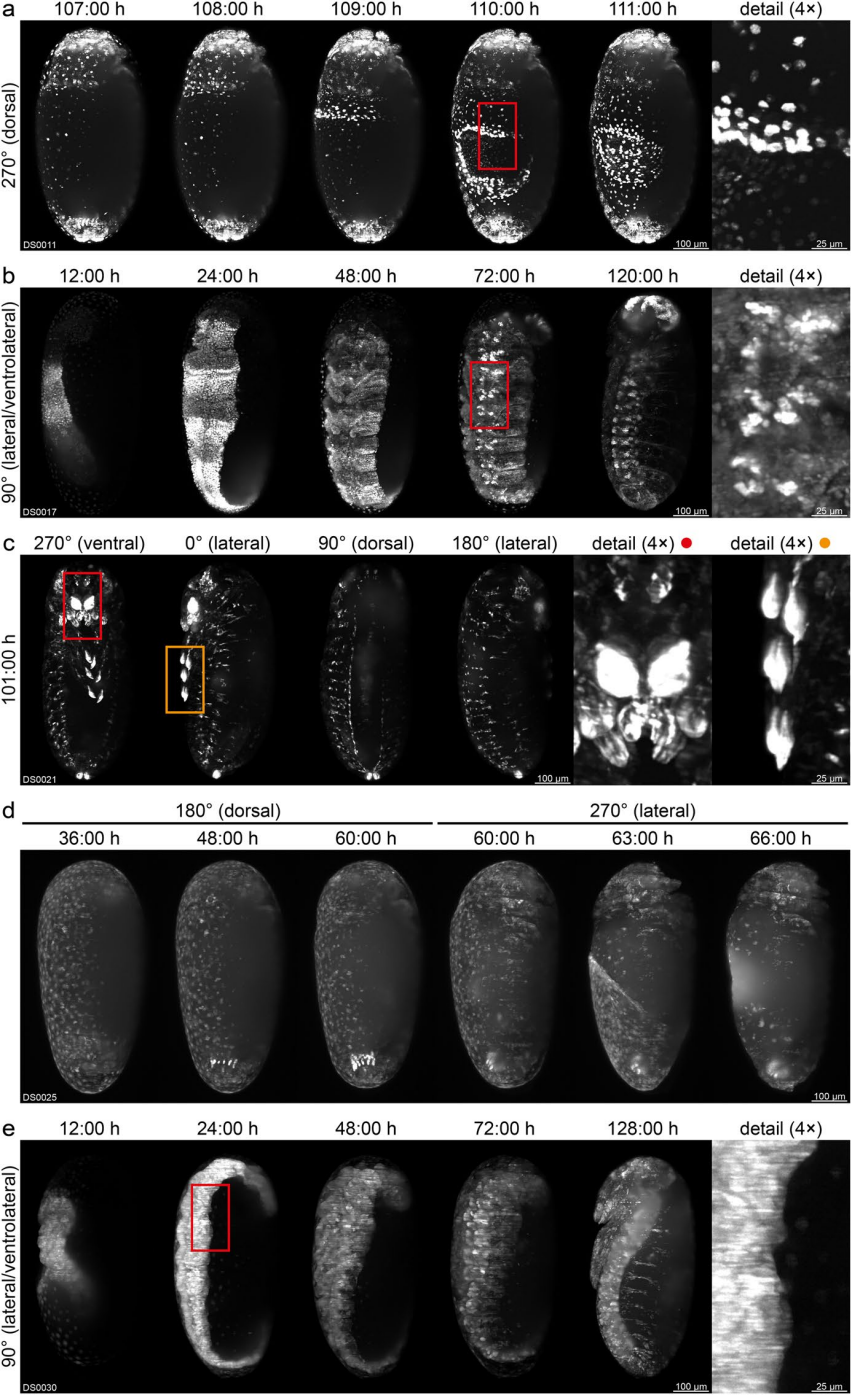
Overview of the expression patterns of the five position-effect sublines. Shown are *z* maximum projections with adjusted brightness and contrast. (**a**) The AGOC{ATub’H2A/H2B°NB-mEmerald} #1 subline shows a weak signal in the serosa during late development, particularly evident during dorsal closure when the amnion and serosa form the dorsal organ. (**b**) The AGOC{ATub’H2A/H2B°NB-mEmerald} #4 subline exhibits strong stripe-like expression in the germband during early development and strong expression in the brain and ventral nerve cord during late development. (**c**) The AGOC{ATub’PCNA°NB-mEmerald} #1 subline displays increasing expression in the head appendages and tarsi during germband retraction, which becomes particularly pronounced after dorsal closure. (**d**) The AGOC{ATub’Lamin°NB-mEmerald} #1 subline shows strong signal in the abdominal tip during germband retraction, which persists through dorsal closure. **(e)** The AGOC{ATub’Lamin°NB-mEmerald} #2 subline exhibits strong, homogeneous signal throughout the embryonic tissue, but only weak signal in the serosa.
- **Confirmation of non-invasive imaging**. To verify that the imaging procedure (e.g. the light exposure) did not induce post-embryonic aberrations, all embryos were retrieved after recording, raised to adulthood, and assessed for fertility. All embryos hatched morphologically intact, except the one from dataset DS0050. Of the resulting larvae, all but the one from DS0026 developed into healthy adults. Among the adults, all except the individual from DS0038 were fertile.

During the entire project and each experiment, particular attention was focused on a systematic approach and dataset comparability. Important technical considerations are:

- **Transgene design**. All newly created sublines are based on highly similar transgenes and all nanobody expression cassettes employ identical promoter and fluorescent protein coding sequences. Further, all transgenes carry the same mOrange- and mCherry-based eye markers. The differences stem from a few nucleotides within the nanobody coding sequences that determine antigen-binding specificity. In consequence, the nanobody-mEmerald fusion proteins are highly similar in terms of amino acid number and sequence. For instance, the histone H2A/H2B and actin nanobodies consist of 125 and 120 amino acids, respectively, and have a pair-wise identity of 68.5%.
- **Nanobody choice**. Nanobodies were chosen according to anticipated utility and availability. Since all nanobodies used in this study were raised against vertebrate antigens, their cross-reactivity with respective *Tribolium* analogues was uncertain. Hence, the cytokeratin-8 nanobody was used as a non-binding nanobody control, since ‘bona fide’ cytoplasmatic intermediate filaments^39^ are not present in *Tribolium* according to genome sequencing and analysis studies^40–42^. Given that future nanobodies will also likely be raised against vertebrate proteins, imaging data from cytokeratin-8 nanobody-expressing sublines provide a valuable reference for assessing binding specificity. For instance, comparison with other sublines (cf. Fig. 1) suggests that nanobodies against histone H2A/H2B and actin bind their respective *Tribolium* orthologues, whereas those against PCNA and lamin A/C do not.
- **Insert number estimation**. Transgenesis via the piggyBac transposase system may result in multiple insertions of the transgene within a single F1 individual^43,44^. In consequence, respective F2 descendants may inherit two or more inserts, leading to variable fluorescence intensity and expression patterns across embryos. To avoid such inconsistencies, the insert number was estimated for each transgenic F2 individual, and only those scoring as single-insert carriers were used to establish sublines (cf. Table 2).
- **Homozygous imaging cultures**. In each newly founded subline, the transgenic F2 individuals as well as their F3 descendants are hemizygous for the transgene. Imaging cultures established using these individuals (e.g. by simply pooling all transgenic F3 descendants) would produce a mixture of wild-type, hemizygous and homozygous embryos, with the latter two likely differing in fluorescence intensity. Hence, for the fifteen sublines confirmed to be homozygous viable, continuative homozygous cultures were established (cf. Supplementary Table 2). These cultures, from which imaging cultures were subsequently drawn, produce exclusively homozygous embryos.
- **Microscope calibration and effective laser power**. Prior to each recording, the DSLM was calibrated using a two-step routine^34^ to avoid common issues (e.g. offset and/or tilt between the light sheet and the focal plane of the detection objective). The effective laser power (i.e. the laser power at the precise location where the embryos were positioned during imaging, as opposed to the nominal output of the laser module) was measured regularly. If deviations were detected, the laser output was adjusted accordingly to ensure consistent illumination light density across all dataset acquisitions.
- **Multiple sublines per transgenic line**. To robustly evaluate the functionality of each of the five nanobodies in *Tribolium* and assess potential inter-subline variability, at least two sublines were created per transgenic line and included in the imaging schedule. This strategy prevents common bottlenecks associated with relying on a single subline such as position effects leading to highly specific expression patterns that may hinder proper evaluation of nanobody functionality (cf. Table 1, ‘PE’ column).
- **Exclusion of sublines from systematic imaging**. Among the six AGOC{ATub’Actin°NB-mEmerald} sublines, only #5 was homozygous viable. In consequence, four sublines (#1, #3, #4, and #6) were completely excluded from imaging, while one subline (#2) was retained to provide morphogenetic data from a second actin nanobody-expressing subline due to a lack of alternatives. Notably, all embryos imaged from this subline were hemizygous for the transgene.
- **Acquisition parameters for systematic imaging**. Embryo collection, preparation, and mounting were standardized across all sublines to ensure consistent imaging conditions. In consequence, recordings were always started at a comparable developmental stage (end of blastoderm formation) and were conducted under identical acquisition parameters (e.g. laser power, exposure time, or temporal interval). Notably, all datasets cover gastrulation (as one of the most dynamic morphogenetic events), and for each subline, at least one dataset was acquired that covers all embryogenetic events from gastrulation to dorsal closure. All recordings were performed on the same microscope, and no major hardware components (e.g. laser module, fluorescence filter, or camera) were replaced during the study period. All embryos were imaged along four directions, capturing ventral, dorsal, and both lateral views (or alternatively both ventrolateral and dorsolateral views). This multi-view approach is particularly important for *Tribolium* embryos, as the germband can rotate around the anterior-posterior axis during development^45^ (cf. Supplementary Table 1, ‘Views’ column).
- **Imaging in triplicates**. To evaluate subline performance and establish a stochastically robust dataset collection, at least three embryos from separate embryo collections were recorded for all sixteen remaining sublines to allow assessment of intra-subline variability. For convenience, replicates were assigned consecutive dataset numbers.
- **Control lines / sublines**. To provide ‘positive control’ data for the histone H2A/H2B and actin nanobody-expressing lines (i.e. those confirmed to bind the respective *Tribolium* orthologue, cf. Figure 1), embryos from three control lines were also imaged. These include the frequently used EFA-nGFP line^30^ (DS0046), which expresses nuclear-localized GFP(S65T) under control of the *elongation factor 1-alpha* promoter, as well as the previously described AGOC{ATub’H2B-mEmerald} #3 and AGOC{ATub’#O(LA)-mEmerald} #1 sublines^23^ (DS0047 and DS0048), which express mEmerald-labeled histone 2B and Lifeact^46^, respectively, under control of the *tubulin alpha 1-like protein* promoter.
- **Identification of position-effect sublines and developmental irregularities**. Comprehensive visual inspection of imaging data revealed that five out of sixteen remaining newly created sublines exhibited position effects (cf. Table 1, ‘PE’ column). A brief overview regarding the expression patterns is provided in Fig. 2. However, for each of the five transgenic lines, at least one subline was obtained that displayed expression as expected, allowing proper evaluation of nanobody performance. Further, three developmental irregularities were observed: Firstly, one embryo from the AGOC{ATub’Lamin°NB-mEmerald} #3 subline exhibited a slight germband torsion, resulting in reduced anteriad (i.e. toward the anterior region of the embryo) movement of the abdominal tip along the dorsal side during germband elongation. Nonetheless, the embryo completed development without further complications (DS0032). Secondly, one embryo from the AGOC{ATub’H2A/H2B°NB-mEmerald} #1 subline exhibited an atypical proliferation pattern during the 12^th^ synchronous division wave of blastoderm formation, but otherwise developed normally (DS0049). As data from this expression-as-expected subline are considered particularly valuable, this dataset was retained in the collection but not counted among the three technical replicates. Finally, embryos from the AGOC{ATub’H2A/H2B°NB-mEmerald} #3 subline frequently displayed developmental aberrations. Hence, only a single exemplary dataset is provided that shows an embryo undergoing self-constriction during dorsal closure (DS0050).

## Usage Notes

The data structure (cf. Data Records section) is compatible with any software capable of handling three-dimensional multi-view dynamic image data. ImageJ^36^ or its derivate Fiji^35^ are recommended as the primary platforms for image processing and analysis. Both are open-source frameworks widely used for working with multi-dimensional microscopy data. In addition, modern community-driven tools such as napari^47^ offer interactive visualization and plugin-based extensibility.

Please note that the file names contain parentheses, which are not part of the Portable Operating System Interface (POSIX) portable filename character set. Therefore, some downstream processing software may require escaping these characters or enclosing the file name in quotation marks. Furthermore, certain operating systems may issue a ‘zip-bomb’ warning when decompressing the archives due to their large size. This can be circumvented by extracting the data using dedicated tools such as 7-Zip or WinRAR, or by temporarily disabling the zip-bomb detection in the native unzip utility by temporarily changing the respective environment variable (export UNZIP_DISABLE_ZIPBOMB_DETECTION = TRUE). The total data volume after extraction exceeds the archive size only minimally.

## Supplementary information

Supplementary Table 1 and 2

## Data Availability

The Zenodo link^38^ redirects to the SLICE-1 dataset collection overview page, which allows entries to be sorted by various convenient criteria. For datasets divided into multiple parts, the corresponding entries are cross-linked in the ‘Additional details’ section. Digital Object Identifiers (DOIs) for all entries are listed in Supplementary Table 1 and can be resolved via the DOI website (https://www.doi.org).

## Code availability

The *Mathematica* program notebook file (.nb) implementing the batch rotation, cropping and zipping procedure is available on GitHub (Strobl2025A-BatchRotCropZIP_V2.5.nb, https://github.com/BugCube/BatchRotCropZIP).
