# Supplementary material for: Fifty high-content light sheet fluorescence microscopy datasets of Tribolium castaneum embryogenesis
Source: Sci Data. 2025 Dec 15;12:1945. doi: 10.1038/s41597-025-06406-6 (PMC12705995; doi:10.1038/s41597-025-06406-6)
Supplement: Supplementary file 1 — Supplementary Table 1 and 2 [file 41597_2025_6406_MOESM1_ESM.pdf]

Supplementary Table 1

Dataset overview with selected metadata. Large datasets are split into multiple parts for convenient download (in such cases, the first part always contains all *t* stack files). DS, dataset; GE, germband elongation; v, ventral; l, lateral; d, dorsal; vl, ventrolateral; dl, dorsolateral. The Digital Object Identifiers (DOIs) can be resolved at https://www.doi.org.

Subline	DS	Total duration / embryogenetic events	Views		Access (DOIs)
			Start of imaging	Transition during GE	
**Datasets from sublines expressing mEmerald-labeled nanobodies against cytokeratin 8**					
AGOC{ATub'Cyto8°NB-mEmerald} #1	0001	24:30 h / blastoderm formation to germband elongation	vl-vl-dl-dl	v-l-d-l	10.5281/zenodo.14605245 (all data)
	0002	24:30 h / blastoderm formation to germband elongation	v-l-d-l	vl-dl-dl-vl	10.5281/zenodo.14605269 (all data)
	0003	121:30 h / blastoderm formation to muscular movement	v-l-d-l	vl-dl-dl-vl	10.5281/zenodo.14605354 (part 1) 10.5281/zenodo.14605412 (part 2)
AGOC{ATub'Cyto8°NB-mEmerald} #2	0004	24:00 h / blastoderm formation to germband elongation	vl-dl-dl-vl	dl-dl-vl-vl	10.5281/zenodo.14605518 (all data)
	0005	24:00 h / blastoderm formation to germband elongation	v-l-d-l	no major change	10.5281/zenodo.14605587 (all data)
	0006	124:00 h / blastoderm formation to muscular movement	vl-dl-dl-vl	no major change	10.5281/zenodo.14650900 (part 1) 10.5281/zenodo.14651042 (part 2)
AGOC{ATub'Cyto8°NB-mEmerald} #3	0007	24:00 h / blastoderm formation to germband elongation	vl-dl-dl-vl	v-l-d-l	10.5281/zenodo.14651120 (all data)
	0008	24:00 h / blastoderm formation to germband elongation	v-l-d-l	vl-dl-dl-vl	10.5281/zenodo.14651190 (all data)
	0009	124:00 h / blastoderm formation to muscular movement	v-l-d-l	no major change	10.5281/zenodo.14651311 (part 1) 10.5281/zenodo.14651388 (part 2)
**Datasets from sublines expressing mEmerald-labeled nanobodies against histone H2A/H2B**					
AGOC{ATub'H2A/H2B°NB-mEmerald} #1	0010	125:00 h / blastoderm formation to muscular movement	v-l-d-l	vl-dl-dl-vl	10.5281/zenodo.14651853 (part 1) 10.5281/zenodo.14652007 (part 2)
	0011	111:00 h / blastoderm formation to dorsal closure	v-l-d-l	l-v-l-d	10.5281/zenodo.14652369 (part 1) 10.5281/zenodo.14652457 (part 2)
	0012	122:00 h / blastoderm formation to muscular movement	vl-dl-dl-vl	l-d-l-v	10.5281/zenodo.14652767 (part 1) 10.5281/zenodo.14652851 (part 2)
AGOC{ATub'H2A/H2B°NB-mEmerald} #2	0013	145:00 h / blastoderm formation to muscular movement	v-l-d-l	no major change	10.5281/zenodo.14653368 (part 1) 10.5281/zenodo.14653663 (part 2)
	0014	119:30 h / blastoderm formation to dorsal closure	v-l-d-l	l-v-l-d	10.5281/zenodo.14654494 (part 1) 10.5281/zenodo.14654893 (part 2)
	0015	124:30 h / early gastrulation to muscular movement	vl-vl-dl-dl	v-l-d-l	10.5281/zenodo.14670925 (part 1) 10.5281/zenodo.14671023 (part 2)
AGOC{ATub'H2A/H2B°NB-mEmerald} #4	0016	142:00 h / early gastrulation to dorsal closure	vl-dl-dl-vl	v-l-d-l	10.5281/zenodo.14671244 (part 1)

					10.5281/zenodo.14671512 (part 2)
	0017	143:00 h / early gastrulation to muscular movement	v-l-d-l	vl-vl-dl-dl	10.5281/zenodo.14671591 (part 1) 10.5281/zenodo.14673530 (part 2)
	0018	143:30 h / early gastrulation to muscular movement	vl-dl-dl-vl	no major change	10.5281/zenodo.14673778 (part 1) 10.5281/zenodo.14673934 (part 2)
**Datasets from sublines expressing mEmerald-labeled nanobodies against PCNA**					
AGOC{ATub'PCNA°NB-mEmerald} #1	0019	24:00 h / blastoderm formation to germband elongation	v-l-d-l	no major change	10.5281/zenodo.14674091 (all data)
	0020	24:00 h / blastoderm formation to germband elongation	v-l-d-l	l-v-l-d	10.5281/zenodo.14674129 (all data)
	0021	125:30 h / early gastrulation to muscular movement	vl-dl-dl-vl	l-d-l-v	10.5281/zenodo.14697086 (part 1) 10.5281/zenodo.14697525 (part 2)
AGOC{ATub'PCNA°NB-mEmerald} #2	0022	64:30 h / blastoderm formation to germband retraction	v-l-d-l	l-v-l-d	10.5281/zenodo.14697679 (all data)
	0023	24:00 h / blastoderm formation to germband elongation	v-l-d-l	l-d-l-v	10.5281/zenodo.14697983 (all data)
	0024	124:30 h / blastoderm formation to muscular movement	v-l-d-l	vl-vl-dl-dl	10.5281/zenodo.14698604 (part 1) 10.5281/zenodo.14698778 (part 2)
**Datasets from sublines expressing mEmerald-labeled nanobodies against lamin A/C**					
AGOC{ATub'Lamin°NB-mEmerald} #1	0025	126:00 h / blastoderm formation to muscular movement	v-l-d-l	no major change	10.5281/zenodo.14699272 (part 1) 10.5281/zenodo.14699380 (part 2)
	0026	126:00 h / blastoderm formation to muscular movement	v-l-d-l	no major change	10.5281/zenodo.14699703 (part 1) 10.5281/zenodo.14699839 (part 2)
	0027	127:00 h / blastoderm formation to muscular movement	vl-dl-dl-vl	no major change	10.5281/zenodo.14700722 (part 1) 10.5281/zenodo.14701852 (part 2)
AGOC{ATub'Lamin°NB-mEmerald} #2	0028	24:00 h / blastoderm formation to germband elongation	v-l-d-l	no major change	10.5281/zenodo.14702621 (all data)
	0029	132:30 h / blastoderm formation to muscular movement	vl-dl-dl-vl	v-l-d-l	10.5281/zenodo.14702997 (part 1) 10.5281/zenodo.14704293 (part 2)
	0030	128:30 h / blastoderm formation to muscular movement	v-l-d-l	vl-vl-dl-dl	10.5281/zenodo.14705721 (part 1) 10.5281/zenodo.14706241 (part 2)
AGOC{ATub'Lamin°NB-mEmerald} #3	0031	24:00 h / blastoderm formation to germband elongation	v-l-d-l	vl-vl-dl-dl	10.5281/zenodo.14711273 (all data)
	0032	128:30 h / blastoderm formation to muscular movement	vl-dl-dl-vl	no major change	10.5281/zenodo.14711536 (part 1) 10.5281/zenodo.14711719 (part 2)
	0033	125:00 h / blastoderm formation to muscular movement	vl-dl-dl-vl	no major change	10.5281/zenodo.14711937 (part 1) 10.5281/zenodo.14712052 (part 2)
AGOC{ATub'Lamin°NB-mEmerald} #4	0034	24:30 h / blastoderm formation to germband elongation	v-l-d-l	vl-dl-dl-vl	10.5281/zenodo.14712312 (all data)
	0035	24:00 h / blastoderm formation to germband elongation	vl-dl-dl-vl	vl-vl-dl-dl	10.5281/zenodo.14712415 (all data)

	0036	128:00 h / blastoderm formation to muscular movement	v-l-d-l	vl-vl-dl-dl	10.5281/zenodo.14712640 (part 1) 10.5281/zenodo.14712743 (part 2)
AGOC{ATub'Lamin°NB-mEmerald} #5	0037	24:30 h / blastoderm formation to germband elongation	v-l-d-l	no major change	10.5281/zenodo.14712950 (all data)
	0038	24:00 h / blastoderm formation to germband elongation	v-l-d-l	l-v-l-d	10.5281/zenodo.14713047 (all data)
	0039	123:30 h / blastoderm formation to muscular movement	v-l-d-l	l-d-l-v	10.5281/zenodo.14713243 (part 1) 10.5281/zenodo.14713342 (part 2)
**Datasets from sublines expressing mEmerald-labeled nanobodies against actin**					
AGOC{ATub'Actin°NB-mEmerald} #2	0040	124:00 h / blastoderm formation to muscular movement	v-l-d-l	no major change	10.5281/zenodo.14713574 (part 1) 10.5281/zenodo.14713671 (part 2)
	0041	141:00 h / early gastrulation to muscular movement	vl-dl-dl-vl	l-d-l-v	10.5281/zenodo.14717217 (part 1) 10.5281/zenodo.14717371 (part 2)
	0042	142:30 h / blastoderm formation to muscular movement	vl-dl-dl-vl	no major change	10.5281/zenodo.14717648 (part 1) 10.5281/zenodo.14717786 (part 2)
AGOC{ATub'Actin°NB-mEmerald} #5	0043	130:00 h / blastoderm formation to muscular movement	v-l-d-l	vl-dl-dl-vl	10.5281/zenodo.14718042 (part 1) 10.5281/zenodo.14718100 (part 2)
	0044	124:30 h / blastoderm formation to muscular movement	vl-vl-dl-dl	v-l-d-l	10.5281/zenodo.14718431 (part 1) 10.5281/zenodo.14718535 (part 2)
	0045	127:30 h / blastoderm formation to muscular movement	vl-dl-dl-vl	v-l-d-l	10.5281/zenodo.14718787 (part 1) 10.5281/zenodo.14718873 (part 2)
**Additional datasets from various lines/sublines (controls and peculiarities of development)**					
EFA-nGFP	0046	142:30 h / blastoderm formation to muscular movement	v-l-d-l	no major change	10.5281/zenodo.14719287 (part 1) 10.5281/zenodo.14720867 (part 2)
AGOC{ATub'H2B-mEmerald} #3	0047	126:00 h / blastoderm formation to muscular movement	v-l-d-l	no major change	10.5281/zenodo.14724054 (part 1) 10.5281/zenodo.14724174 (part 2)
AGOC{ATub'#O(LA)-mEmerald} #1	0048	150:00 h / early gastrulation to muscular movement	v-l-d-l	l-v-l-d	10.5281/zenodo.14724769 (part 1) 10.5281/zenodo.14724839 (part 2) 10.5281/zenodo.14725099 (part 3) 10.5281/zenodo.14725153 (part 4)
AGOC{ATub'H2A/H2B°NB-mEmerald} #1	0049	94:30 h / blastoderm formation to germband retraction	vl-dl-dl-vl	v-l-d-l	10.5281/zenodo.14725315 (part 1) 10.5281/zenodo.14725315 (part 2)
AGOC{ATub'H2A/H2B°NB-mEmerald} #3	0050	124:00 h / blastoderm formation to dorsal closure	v-l-d-l	no major change	10.5281/zenodo.14725492 (part 1) 10.5281/zenodo.14725694 (part 2)

Supplementary Table 2

AGOC-associated mating procedure results for a systematic creation of homozygous cultures. For a detailed description of the mating procedure, please refer to the respective study[1]. Bold entries mark progeny that were used in the subsequent cross or to establish F7+ continuative homozygous cultures (F7 progeny). F6-S, F7-O and F7-C are control crosses. Homozygous F7 individuals were obtained for fifteen of the twenty sublines.

Gen	Cross	Subline		Progeny								
				⚫⚫⚫	🔵⚫⚫	⚫🟠⚫	⚫⚫🔴	🔵🟠⚫	🔵⚫🔴	⚫🟠🔴	🔵🟠🔴	Total
F3	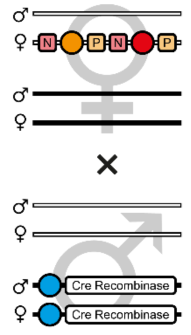	Theoretical	-	-	50.0%	-	-	-	-	-	50.0%	-
		AGOC{ATub'Cyto8°NB-mEmerald}	#1	-	53.5% (68)	-	-	-	-	-	**46.5% (59)**	127
			#2	-	50.9% (59)	-	-	-	-	-	**49.1% (57)**	116
			#3	-	57.3% (51)	-	-	-	-	-	**42.7% (38)**	89
		AGOC{ATub'H2A/H2B°NB-mEmerald}	#1	-	41.8% (46)	-	-	-	-	-	**58.2% (64)**	110
			#2	-	39.1% (9)	-	-	-	-	-	**60.9% (14)**	23
			#3	-	48.2% (41)	-	-	-	-	-	**51.8% (44)**	85
			#4	-	58.0% (40)	-	-	-	-	-	**42.0% (29)**	69
		AGOC{ATub'PCNA°NB-mEmerald}	#1	-	50.0% (53)	-	-	-	-	-	**50.0% (53)**	106
			#2	-	47.1% (49)	-	-	-	-	-	**52.9% (55)**	104
		AGOC{ATub'Lamin°NB-mEmerald}	#1	-	38.3% (36)	-	-	-	-	-	**61.7% (58)**	94
			#2	-	49.3% (68)	-	-	-	-	-	**50.7% (70)**	138
			#3	-	50.4% (64)	-	-	-	-	-	**49.6% (63)**	127
			#4	-	53.5% (54)	-	-	-	-	-	**46.5% (47)**	101
			#5	-	53.3% (64)	-	-	-	-	-	**46.7% (56)**	120
		AGOC{ATub'Actin°NB-mEmerald}	#1	-	48.0% (48)	-	-	-	-	-	**52.0% (52)**	100
			#2	-	51.3% (60)	-	-	-	-	-	**48.7% (57)**	117
			#3	-	53.5% (69)	-	-	-	-	-	**46.5% (60)**	129
			#4	-	50.0% (55)	-	-	-	-	-	**50.0% (55)**	110
			#5	-	54.3% (44)	-	-	-	-	-	**45.7% (37)**	81
			#6	-	41.3% (31)	-	-	-	-	-	**58.7% (44)**	75
		Mean ± SD	-	-	49.5% ± 5.6%	-	-	-	-	-	50.5% ± 5.6%	101.1 ± 26.3
F4	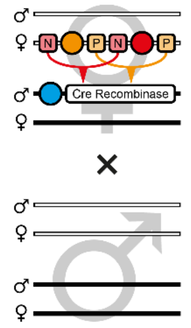	Theoretical	-	25.0%	25.0%	12.5%	12.5%	12.5%	12.5%	-	-	-
		AGOC{ATub'Cyto8°NB-mEmerald}	#1	20.5% (27)	38.7% (51)	**12.1% (16)**	**8.3% (11)**	12.1% (16)	8.3% (11)	-	-	132
			#2	18.3% (21)	40.9% (47)	**6.2% (7)**	**6.9% (8)**	11.3% (13)	16.5% (19)	-	-	115
			#3	20.0% (17)	28.2% (24)	**21.2% (18)**	**4.7% (4)**	22.4% (19)	3.5% (3)	-	-	85
		AGOC{ATub'H2A/H2B°NB-mEmerald}	#1	12.2% (10)	46.3% (38)	**8.5% (7)**	**2.5% (2)**	25.6% (21)	4.9% (4)	-	-	82
			#2	35.5% (39)	20.9% (23)	**8.2% (9)**	**12.7% (14)**	18.2% (20)	4.5% (5)	-	-	110
			#3	15.7% (8)	27.4% (14)	**11.8% (6)**	**11.8% (6)**	19.6% (10)	13.7% (7)	-	-	51
			#4	37.9% (42)	18.0% (20)	**14.4% (16)**	**9.9% (11)**	17.1% (19)	2.7% (3)	-	-	111
		AGOC{ATub'PCNA°NB-mEmerald}	#1	29.0% (27)	21.5% (20)	**19.4% (18)**	**5.4% (5)**	18.3% (17)	6.4% (6)	-	-	93
			#2	13.5% (22)	44.8% (73)	**6.1% (10)**	**1.8% (3)**	20.9% (34)	12.9% (21)	-	-	163
		AGOC{ATub'Lamin°NB-mEmerald}	#1	18.8% (13)	47.8% (33)	**7.3% (5)**	**4.4% (3)**	8.7% (5)	13.0% (9)	-	-	69
			#2	17.5% (7)	22.5% (9)	**25.0% (10)**	**10.0% (4)**	5.0% (2)	20.0% (8)	-	-	40
			#3	31.6% (35)	30.6% (34)	**16.2% (18)**	**4.5% (5)**	8.1% (9)	9.0% (10)	-	-	111
			#4[2]	8.3% (6)	38.9% (28)	0.0% (0)	**6.9% (5)**	**1.4% (1)**	1.4% (1)	-	43.1% (31)	72
			#5	23.4% (22)	27.6% (26)	**11.7% (11)**	**9.6% (9)**	12.8% (12)	14.9% (14)	-	-	94
		AGOC{ATub'Actin°NB-mEmerald}	#1	21.5% (23)	24.3% (26)	**11.2% (12)**	**14.0% (15)**	16.8% (18)	12.2% (13)	-	-	107
			#2	22.5% (23)	24.5% (25)	**17.6% (18)**	**2.0% (2)**	31.4% (32)	2.0% (2)	-	-	102
			#3	5.1% (6)	41.5% (49)	**34.7% (41)**	**5.9% (7)**	7.6% (9)	4.2% (5)	-	-	118
			#4	16.3% (13)	32.5% (26)	**26.2% (21)**	**2.5% (2)**	21.2% (17)	1.3% (1)	-	-	80
			#5	25.8% (24)	28.0% (26)	**19.4% (18)**	**3.2% (3)**	20.4% (19)	3.2% (3)	-	-	93
			#6	22.6% (27)	26.1% (31)	**11.8% (14)**	**15.1% (18)**	12.6% (15)	11.8% (14)	-	-	119
		Mean ± standard deviation	-	21.5% ± 8.0%	31.2% ± 9.3%	15.2% ± 7.7%	7.1% ± 4.6%	16.3% ± 6.8%	8.7% ± 5.6%	-	-	98.7 ± 28.2
F5	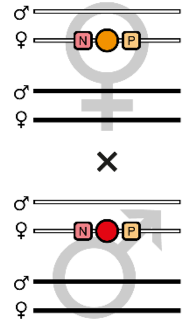	Theoretical	-	25.0%	-	25.0%	25.0%	-	-	25.0%	-	-
		AGOC{ATub'Cyto8°NB-mEmerald}	#1	31.8% (42)	-	21.2% (28)	22.7% (30)	-	-	**24.3% (32)**	-	132
			#2	29.4% (20)	-	27.9% (19)	17.7% (12)	-	-	**25.0% (17)**	-	68
			#3	29.5% (26)	-	29.5% (26)	14.8% (13)	-	-	**26.2% (23)**	-	88
		AGOC{ATub'H2A/H2B°NB-mEmerald}	#1	4.5% (5)	-	41.5% (46)	33.3% (37)	-	-	**20.7% (23)**	-	111
			#2	26.2% (21)	-	25.0% (20)	27.5% (22)	-	-	**21.3% (17)**	-	80
			#3	21.8% (24)	-	21.8% (24)	28.2% (31)	-	-	**28.2% (31)**	-	110
			#4	27.4% (31)	-	25.7% (29)	18.6% (21)	-	-	**28.3% (32)**	-	113
		AGOC{ATub'PCNA°NB-mEmerald}	#1	26.4% (29)	-	31.8% (35)	21.8% (24)	-	-	**20.0% (22)**	-	110
			#2	31.2% (44)	-	25.5% (36)	21.3% (30)	-	-	**22.0% (31)**	-	141
		AGOC{ATub'Lamin°NB-mEmerald}	#1	39.4% (13)	-	24.2% (8)	12.2% (4)	-	-	**24.2% (8)**	-	33
			#2	35.2% (25)	-	23.9% (17)	28.2% (20)	-	-	**12.7% (9)**	-	71
			#3	35.1% (20)	-	24.6% (14)	14.0% (8)	-	-	**26.3% (15)**	-	57
			#4[2]	14.4% (14)	18.6% (18)	10.3% (10)	9.3% (9)	11.3% (11)	11.3% (11)	**16.5% (16)**	8.3% (8)	97
			#5	37.5% (18)	-	22.9% (11)	20.8% (10)	-	-	18.8% (9)	-	48
		AGOC{ATub'Actin°NB-mEmerald}	#1[3]	33.3% (5)	-	40.0% (6)	26.7% (4)	-	-	0.0% (0)	-	15
			#2[3]	39.5% (15)	-	21.1% (8)	28.9% (11)	-	-	10.5% (4)	-	38
			#3[3]	22.7% (5)	-	22.7% (5)	36.4% (8)	-	-	18.2% (4)	-	22
			#4[3]	39.0% (16)	-	24.4% (10)	29.3% (12)	-	-	7.3% (3)	-	41
			#5	30.6% (45)	-	23.8% (35)	25.2% (37)	-	-	**20.4% (30)**	-	147
			#6[3]	29.6% (8)	-	33.4% (9)	25.9% (7)	-	-	11.1% (3)	-	27
		Mean ± standard deviation	-	30.0% ± 8.1%	-	26.9% ± 5.9%	23.9% ± 6.5%	-	-	19.2% ± 7.7%	-	76.4 ± 42.3

F6	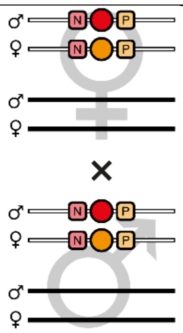	Theoretical	-	-	-	25.0%	25.0%	-	-	50.0%	-	-
		AGOC{ATub'Cyto8°NB-mEmerald}	#1	-	-	**24.0% (25)**	**25.0% (26)**	-	-	51.0% (53)	-	104
			#2	-	-	**30.0% (15)**	**24.0% (12)**	-	-	46.0% (23)	-	50
			#3	-	-	**36.1% (31)**	**24.4% (21)**	-	-	39.5% (34)	-	86
		AGOC{ATub'H2A/H2B°NB-mEmerald}	#1	-	-	**21.3% (17)**	**22.5% (18)**	-	-	56.2% (45)	-	80
			#2	-	-	**23.7% (19)**	**21.3% (17)**	-	-	55.0% (44)	-	80
			#3	-	-	**19.7% (14)**	**31.0% (22)**	-	-	49.3% (35)	-	71
			#4	-	-	**28.4% (39)**	**21.2% (29)**	-	-	50.4% (69)	-	137
		AGOC{ATub'PCNA°NB-mEmerald}	#1	-	-	**28.6% (22)**	**16.9% (13)**	-	-	54.5% (42)	-	77
			#2	-	-	**21.7% (23)**	**31.1% (33)**	-	-	47.2% (50)	-	106
		AGOC{ATub'Lamin°NB-mEmerald}	#1	-	-	**18.9% (23)**	**29.5% (36)**	-	-	47.6% (58)	-	122
			#2	-	-	**23.1% (15)**	**24.6% (16)**	-	-	52.3% (34)	-	65
			#3	-	-	**21.2% (14)**	**33.3% (22)**	-	-	45.5% (30)	-	66
			#4	-	-	**26.3% (36)**	**29.9% (41)**	-	-	43.8% (60)	-	137
			#5	-	-	**13.1% (6)**	**21.7% (10)**	-	-	65.2% (30)	-	46
		AGOC{ATub'Actin°NB-mEmerald}	#1	not assayed since the F5 cross resulted in inconvenient progeny								
			#2	not assayed since the F5 cross resulted in inconvenient progeny								
			#3	not assayed since the F5 cross resulted in inconvenient progeny								
			#4	not assayed since the F5 cross resulted in inconvenient progeny								
			#5	-	-	**32.9% (29)**	**20.5% (18)**	-	-	20.4% (30)	-	88
			#6	not assayed since the F5 cross resulted in inconvenient progeny								
		Mean ± standard deviation	-	-	-	24.6% ± 5.9%	25.1% ± 4.8%	-	-	48.3% ± 9.8%	-	87.7
F6-S	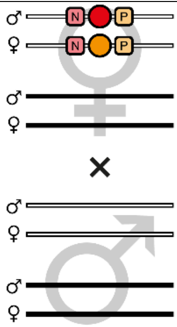	Theoretical	-	-	-	50.0%	50.0%	-	-	-	-	-
		AGOC{ATub'Cyto8°NB-mEmerald}	#1	-	-	48.4% (59)	51.6% (63)	-	-	-	-	122
			#2	-	-	56.1% (55)	43.9% (43)	-	-	-	-	98
			#3	-	-	46.0% (52)	54.0% (61)	-	-	-	-	113
		AGOC{ATub'H2A/H2B°NB-mEmerald}	#1	-	-	52.1% (61)	47.9% (56)	-	-	-	-	117
			#2	-	-	23.7% (19)	21.3% (17)	-	-	-	-	37
			#3	-	-	54.5% (61)	45.5% (51)	-	-	-	-	112
			#4	-	-	44.6% (45)	55.4% (56)	-	-	-	-	101
		AGOC{ATub'PCNA°NB-mEmerald}	#1	-	-	48.5% (66)	51.5% (70)	-	-	-	-	136
			#2	-	-	53.2% (42)	46.8% (37)	-	-	-	-	79
		AGOC{ATub'Lamin°NB-mEmerald}	#1	-	-	52.1% (62)	47.9% (57)	-	-	-	-	119
			#2	-	-	54.4% (56)	45.6% (47)	-	-	-	-	103
			#3	-	-	48.2% (56)	51.8% (71)	-	-	-	-	127
			#4	-	-	51.2% (63)	48.8% (60)	-	-	-	-	123
			#5	-	-	47.9% (46)	52.1% (50)	-	-	-	-	96
		AGOC{ATub'Actin°NB-mEmerald}	#1	see entry in the respective F6 row								
			#2	see entry in the respective F6 row								
			#3	see entry in the respective F6 row								
			#4	see entry in the respective F6 row								
			#5	-	-	52.6% (61)	47.4% (55)	-	-	-	-	116
			#6	see entry in the respective F6 row								
		Mean ± standard deviation	-	-	-	50.8% ± 3.4%	49.2% ± 3.4%	-	-	-	-	106.5
F7-O	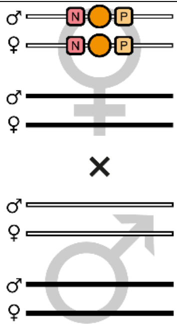	Theoretical	-	-	-	100%	-	-	-	-	-	-
		AGOC{ATub'Cyto8°NB-mEmerald}	#1	-	-	100% (107)	-	-	-	-	-	107
			#2	-	-	100% (72)	-	-	-	-	-	72
			#3	-	-	100% (93)	-	-	-	-	-	93
		AGOC{ATub'H2A/H2B°NB-mEmerald}	#1	-	-	100% (98)	-	-	-	-	-	98
			#2	-	-	100% (103)	-	-	-	-	-	103
			#3	-	-	100% (68)	-	-	-	-	-	68
			#4	-	-	100% (89)	-	-	-	-	-	89
		AGOC{ATub'PCNA°NB-mEmerald}	#1	-	-	100% (107)	-	-	-	-	-	107
			#2	-	-	100% (98)	-	-	-	-	-	98
		AGOC{ATub'Lamin°NB-mEmerald}	#1	-	-	100% (101)	-	-	-	-	-	101
			#2	-	-	100% (85)	-	-	-	-	-	85
			#3	-	-	100% (94)	-	-	-	-	-	94
			#4	-	-	100% (102)	-	-	-	-	-	102
			#5	-	-	100% (77)	-	-	-	-	-	77
		AGOC{ATub'Actin°NB-mEmerald}	#1	see entry in the respective F6 row								
			#2	see entry in the respective F6 row								
			#3	see entry in the respective F6 row								
			#4	see entry in the respective F6 row								
			#5	-	-	100% (81)	-	-	-	-	-	81
			#6	see entry in the respective F6 row								
		Mean ± standard deviation	-	-	-	100% ± 0%	-	-	-	-	-	91.7 ± 12.6
F7-C	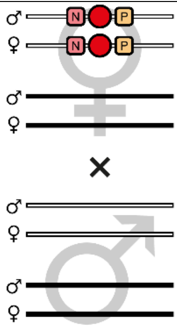	Theoretical	-	-	-	-	100%	-	-	-	-	-
		AGOC{ATub'Cyto8°NB-mEmerald}	#1	-	-	-	100% (59)	-	-	-	-	59
			#2	-	-	-	100% (76)	-	-	-	-	76
			#3	-	-	-	100% (66)	-	-	-	-	66
		AGOC{ATub'H2A/H2B°NB-mEmerald}	#1	-	-	-	100% (67)	-	-	-	-	67
			#2	-	-	-	100% (77)	-	-	-	-	77
			#3	-	-	-	100% (99)	-	-	-	-	99
			#4	-	-	-	100% (56)	-	-	-	-	56
		AGOC{ATub'PCNA°NB-mEmerald}	#1	-	-	-	100% (62)	-	-	-	-	62
			#2	-	-	-	100% (166)	-	-	-	-	166
		AGOC{ATub'Lamin°NB-mEmerald}	#1	-	-	-	100% (78)	-	-	-	-	78
			#2	-	-	-	100% (68)	-	-	-	-	68
			#3	-	-	-	100% (122)	-	-	-	-	122
			#4	-	-	-	100% (78)	-	-	-	-	78
			#5	-	-	-	100% (118)	-	-	-	-	118
		AGOC{ATub'Actin°NB-mEmerald}	#1	see entry in the respective F6 row								
			#2	see entry in the respective F6 row								
			#3	see entry in the respective F6 row								
			#4	see entry in the respective F6 row								
			#5	-	-	-	100% (59)	-	-	-	-	59
			#6	see entry in the respective F6 row								
		Mean ± standard deviation	-	-	-	-	100% ± 0%	-	-	-	-	83.4 ± 30.6

[1]Strobl, F., Anderl, A. & Stelzer, E. H. K. A universal vector concept for a direct genotyping of transgenic organisms and a systematic creation of homozygous lines. *eLife* **7**, e31677 (2018). https://doi.org/10.7554/eLife.31677 [2]In the AGOC{ATub'Lamin°NB-mEmerald} #4 subline, incomplete recombination occurred in the F4 (mCe; mO-mC) double hemizygous generation, as we obtained several F5 individuals that still carried both transformation markers. Due to a lack of F5 (mO) post-recombination hemizygous descendants, we continued the mating procedure with a F5 (mCe; mO) double hemizygous individual, which resulted in an atypical progeny distribution in the F6 generation. However, (mO/mC) heterozygous descendants required for the continuation of the crossing procedure were found (as expected). The subline was excluded from the F5 and F6 mean calculations. [3]The F5 cross of the AGOC{ATub'Actin°NB-mEmerald} #1, #2, #3, #4, and #6 sublines resulted in only very low progeny numbers, and the few (mO/mC) heterozygous descendants died before developing into an adult.
